# Geobotanical Study and Preservation of Rare and Endangered Rosaceae Species

**DOI:** 10.3390/plants14101526

**Published:** 2025-05-19

**Authors:** Natalya V. Romadanova, Alina S. Zemtsova, Nazira A. Altayeva, Natalya A. Artimovich, Alyona M. Alexandrova, Svetlana V. Kushnarenko, Jean Carlos Bettoni

**Affiliations:** 1 Institute of Plant Biology and Biotechnology, 45 Timiryazev St., Almaty 050040, Kazakhstan; daizy-c@mail.ru (N.A.A.); artimovich_nata@mail.ru (N.A.A.); sv.kushnarenko.bio@gmail.com (S.V.K.); 2 M. A. Aitkhozhin Institute of Molecular Biology and Biochemistry, Almaty 050012, Kazakhstan; a.alexandrova@imbb.org.kz; 3 The New Zealand Institute for Plant and Food Research Limited, Canterbury Agriculture & Science Centre, 74 Gerald St, Lincoln 7608, New Zealand

**Keywords:** cryopreservation, genetic diversity, in vitro collection, plant tissue culture, wild species

## Abstract

The loss of plant species, especially endangered and endemic ones, poses a significant threat to global biodiversity. These species cannot easily be replaced when their populations decline or become extinct, which makes their loss particularly devastating. This study focuses on the geobotanical study of nine Rosaceae species (*Cotoneaster karatavicus*, *Crataegus ambigua*, *Malus niedzwetzkyana*, *Malus sieversii*, *Prunus tenella*, *Prunus ulmifolia*, *Sibiraea laevigata*, *Sorbus persica*, and *Spiraeanthus schrenkianus*) and the development of ex situ approaches for the conservation of Rosaceae species listed in the Red Book of Kazakhstan. The geobotanical study revealed an alarming trend of biodiversity loss in five regions of Kazakhstan. This loss is driven by threats from diseases and pests, as well as the aging of plants, small population sizes, weak in situ fruiting, and other factors, such as climate change. We have established an in vitro collection for the short- and medium-term conservation of seeds, embryos and shoots taken either directly from field-grown plants or from budwood cuttings forced indoors. We also use long-term sexual conservation methods, such as the cryopreservation of seed and embryonic axes, alongside conventional seed banking at −20 °C. Ex situ conservation efforts that use multiple propagules and storage methods for the same species are well-suited to a diverse genebank facility. These efforts enable future generations to use this valuable reservoir of genetic diversity for crop improvement and may also serve as a basis for propagating planting material to restore degraded populations.

## 1. Introduction

Given the alarming rates of biodiversity loss and the significant threat of extinction facing many plant species, there has been an increasing focus on the conservation and safeguarding of rare and endangered flora [1,2]. These verdant treasures of our planet harbor unique genetic codes and play an indispensable role in their ecosystems as key sources of genetic material. Efforts are urgently needed to preserve and restore dwindling populations for the long-term sustainability of agriculture [3,4,5,6]. Wild Rosaceae species offer many desirable traits, such as resistance to pathogens and tolerance to abiotic stress. Therefore, conserving this genetic diversity is critical to ensure continued access to these potentially valuable traits for future breeding efforts and improved crop production [7,8,9,10,11,12,13].

The Rosaceae family is a diverse and economically significant group of plants comprising over 3000 species ranging from ornamental shrubs to fruit-bearing trees. The diverse morphological and ecological traits of the Rosaceae family contribute to overall biodiversity by supporting a wide range of species and ecosystem functions, as well as offering valuable traits for crop improvement. Many of these species around the world are now at risk of extinction due to a combination of escalating human activities and natural events [12,14,15]. Kazakhstan, with its rich tapestry of natural landscapes and unparalleled botanical diversity [16,17,18], is no exception to this alarming trend. Within its borders, 13 species of the Rosaceae family are considered endangered, earning them a place in the Red Book of Kazakhstan: *Cotoneaster karatavicus* Pojark. (endemic); *Crataegus ambigua* C.A.Mey. ex A.K.Becker; *Drymocallis tianschanica* (TH.Wolf) Soják; *Malus niedzwetzkyana* Dieck ex Koehne (relict); *M. sieversii* (Ledeb.) M. Roem. (relict); *Prunus armeniaca* L.; *Prunus tenella* Batsch (endemic); *Prunus ulmifolia* (Franch.) (relict); *Rosa cinnamomea* L. (endemic); *Sibiraea laevigata* (L.) Maxim.; *Sibiraea tianschanica* Pojark (endemic); *Sorbus persica* Hedl. and *Spiraeanthus schrenkianus* (Fisch. & C.A.Mey.) Maxim. (endemic) [19]. The genetic pool of these taxa is at risk of loss in the wild. Thus, the collection of specimens and the development of conservation strategies ex situ are urgent [20].

Rosaceae breeding programs require access to high-quality plant material, including that from wild species found in their native in situ locations, such as some areas in Kazakhstan [8,17,21,22,23]. Traditionally, Rosaceae genebanks are maintained as living trees in field collections [18,24,25,26]. The establishment of duplicate collections in a secondary field or other ex situ strategy is a commendable strategy to minimize the risk of loss from attacks by pests and diseases, ageing of the plants, and environmental diseases [27,28,29,30,31,32,33].

Ex situ conservation approaches including duplicates in vitro and cryopreservation have been used to safeguard valuable and vulnerable plant genetic resources within genebanks, including Rosaceae species [9,27,33,34]. In vitro genebanks provide an alternative to field collections for the short- and medium-term storage of plant genetic resources and are commonly used for massive micropropagation [27,35,36,37,38,39,40]. Cryopreservation, the storage of biological materials in liquid nitrogen (LN, −196 °C), is a safe and cost-effective strategy for the long-term storage of plant genetic resources and complements traditional field and in vitro collections [30,33,41,42,43,44,45]. These ex situ conservation methods, alongside in situ collections, provide a robust framework for the enduring protection of our botanical heritage [46].

Successful international examples of the use of these in vitro biotechnologies for the conservation of plant genetic resources, especially for endangered species, provide valuable lessons for Kazakhstan [41,47,48,49,50]. The focus of the Institute of Plant Biology and Biotechnology in Almaty is to conserve genetic diversity, in the form of alleles, and specific traits, as well as specific genetic combinations (clones) that may be of key interest to the breeding and research programs. In vitro collections, established from vegetatively propagated material, are aimed at the conservation of clones in which specific genetic combinations are preserved [30,49]. Tissue-cultured plants are maintained under standard tissue culture or slow growth conditions (reduced temperature, low light, modified media) [36,38,40]. In addition, plant material maintained in vitro can serve as a year-round source for clonal cryopreservation when it is better suited to the program’s conservation goals, as well as for disease eradication and the exchange of plant genetic resources [31,32,33,34,51]. Conserving propagules, such as seeds, allows for the capture of a larger portion of population diversity than clones, and it is therefore a more suitable source of propagules for conserving wild species. Seeds and seed-derived embryonic axes have also been used as cryopreserved propagules within genebanks. Therefore, the choice of propagules to be cryopreserved is highly dependent on the desired conservation targets (e.g., genes or specific allelic combinations) of the genebanks [30,49,52]. These conservation efforts require, to some extent, the use of aseptic in vitro cultures, and therefore the development of successful in vitro culture systems is critical. In vitro culture systems should be optimized to ensure that cultures are clean and that optimal culture media formulations have been established, which can vary from species to species and often between cultivars of the same species, especially wild species [30]. This study focuses on the geobotanical study of nine Rosaceae species (*C. karatavicus*, *C. ambigua*, *M. niedzwetzkyana*, *M. sieversii*, *P. tenella*, *P. ulmifolia*, *S. laevigata*, *S. persica*, and *S. schrenkianus*) and the development of ex situ approaches for the conservation of Rosaceae species listed in the Red Book of Kazakhstan, which are now at risk of being lost due to climate change and habitat destruction. We have established an in vitro clonal collection for short- and medium-term conservation, as well as long-term sexual conservation using seed and embryonic axes cryopreservation. These valuable reservoirs of genetic diversity are now available to support current and future efforts in research and breeding programs aimed at improving crops.

## 2. Results and Discussion

### 2.1. Description of the Rosaceae Species Cultivated in Their Natural Habitat

**Figure 1:**
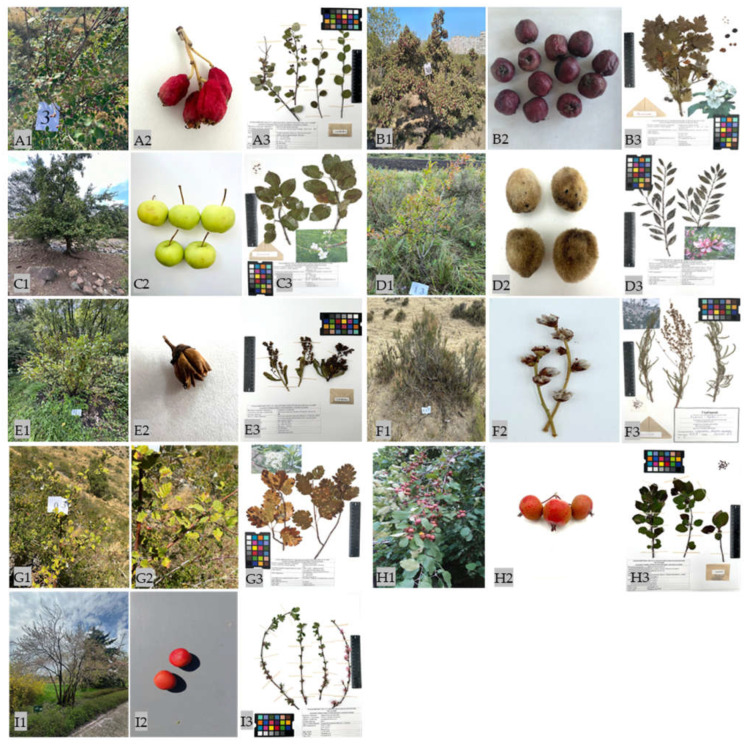
Natural populations of Rosaceae species conserved in in situ conservation sites in Kazakhstan, their fruit appearance and herbarium collections. (**A1**), (**A2**), and (**A3**): *Cotoneaster karatavicus*; (**B1**), (**B2**), and (**B3**): *Crataegus ambigua*; (**C1**), (**C2**), and (**C3**): *Malus sieversii*; (**D1**), (**D2**), and (**D3**): *Prunus tenella*; (**E1**), (**E2**), and (**E3**): *Sibiraea laevigata*; (**F1**), (**F2**), and (**F3**): *Spiraeanthus schrenckianus*; (**G1**), (**G2**), and (**G3**): *Sorbus persica;* (**H1**), (**H2**), and (**H3**): *M. niedzwetzkyana*; (**I1**), (**I2**), and (**I3**)**:** *P. ulmifolia*.

Kazakhstan has rich resources of plant genetic diversity, including cultivated and wild Rosaceae species [20,53,54]. Some of these Rosaceae species are uniquely represented by plants that grow in their natural habitats. These valuable accessions display a wide range of phenotypic and genetic diversity, which is essential for further crop improvement. Given the ongoing disruption of their natural habitats, some of these plants are listed in the Red Book of Kazakhstan, which increases the risk of genetic erosion. The distribution area of the Red Book Rosaceae plants in Kazakhstan covers seven regions from the Caspian Sea to the Altai Mountains, regions with such a diverse and rich flora [19,53]. Following expeditions to different regions, a wide range of plant morphology was observed among nine Rosaceae species, from 40 cm semi-shrubs (*S. laevigata*) to 5–8 m trees (*M. sieversii*). The collections made during the 2023–2024 expeditions included 250 accessions representing nine Rosaceae species. These species were found in their natural habitats, primarily on rocky slopes at altitudes ranging from 4 to 1883 m above sea level. Figure 1 shows the general appearance of natural populations of Rosaceae species in in situ conservation sites in Kazakhstan, as well as their fruits and herbarium. The Rosaceae species generally exhibited weak in situ fruiting, with a varied spectrum of fruit types that differed in size and color. These fruit types were mainly apical. All plant accessions that provided material during the expeditions were characterized and numbered. Nine species were then described according to the descriptors. The characterization of the different species and their surrounding species is described below. The distribution of the key qualitative morphological features among the different Rosaceae species is presented in Supplementary Table S1.

Three populations of *Cotoneaster karatavicus* were identified. They consisted mainly of viable, evergreen adults, about 1.5 m in height with clusters of small red berries. The populations were noted to have low susceptibility to diseases and low incidence of pests, and the main vegetation surrounding them was *Acer platanoides* L., *C. laevigata* (Poir.) DC., *Fraxinus excelsior* L., and *Rosa spinosissima* L.

Four populations of *C. ambigua* were found. They were mainly represented by viable, adult, fruit-bearing shrubs that were about 3–4 m high and had reddish fruits. The four populations were highly susceptible to insects, mainly apple aphid (*Aphis pomi* De Geer), consuming more than 50% of the leaf mass. The dominant vegetation at the collection site consisted primarily of *Caragana grandiflora* (M. Bieb.) DC., *Elaeagnus angustifolia* L., *Morus alba* L., and *Rhamnus pallasii* ssp. *sintenisii* (Rech. Fil.).

Only three apple trees specimens of *M. niedzwetzkyana*, representing two populations, were found in two locations. Two of the trees were found in the Kyrgyzsai Gorge (population 1). One of the trees was young and fruitful (red/pink-fleshed apples), while the other was an adult, diseased tree that did not produce fruit. During the expeditions in the Ili District (population 2), one tree was found and consisted of a viable young and fruitful tree with red/pink-fleshed apples. All three trees were smaller than 5 m tall. In population 1, both trees had high levels of insect infestation and fire blight (caused by a Gram-negative bacterium *Erwinia amylovora*). In contrast, population 2 had lower levels of both insect infestation and disease infection. The main pests identified in both populations were *Cydia pomonella* (Linnaeus), *Hyphantria cunea* Drury., and *Tetranychidae Donnadieu*, and the surrounding plant species were *Berberis heteropoda* Schrenk, *P. armeniaca* L., and *Ulmus pumila* L.

Three populations of *M. sieversii* were found. They consisted mainly of mature, viable trees (57.1%), as well as young, fruit-bearing trees with green and yellowish-green fruits (14.3%). The populations also included old, decrepit trees (8.6%) and mature, diseased trees (8.6%). The trees were primarily medium-height (5–7 m, 45.7%) and tall trees (>7 m, 40.0%), while low-growing trees (<5 m) were less common (14.3%). The incidence of diseased trees was low and primarily caused by *Venturia pirina*, *Plasmopara viticola* and unidentified rust diseases. The predominant plant species around the collection site were *B. heteropoda* Schrenk, *Echinacea angustifolia*, *Lonicera tatarica* L., and *P. armeniaca* L.

Two populations of *P. tenella*, commonly known as the Russian dwarf almond, were found. The populations consisted mostly of viable, non-fruiting adult shrubs (35.0%) and fruit-bearing shrubs (35.0%). A smaller number of accessions were young, non-fruiting shrubs (15.0%) and fruit-bearing shrubs (15.0%). The average height of the shrubs was between 1 and 1.5 m, and their almond-like fruits were drupes covered with dull, velvety yellow skin. Interestingly, no pests or diseases were identified, and the surrounding plant species at the collection sites were *L. tatarica*, *Rose spinosissima*, *Spiraea hypericifolia*, and *Stipa capillata* L.

Only one population of *P. ulmifolia* was found in the territory of the main Botanical Garden of Almaty. This species was not found during the expedition to other places. The accessions consisted of 53.3% viable adult and 46.7% young fruiting shrubs, which had an average height of about 3 m. Weak fruiting was observed, and the fruits were reddish drupes. No pests or diseases were detected. This population was collected in the Botanical Garden in Almaty. Other species growing around the site collection included *Thuja standishii* (Gordon) Carrière, *T. koraiensis* Nakai, *Corylus avellana* (L.) H. Karst., *Crataegus almaatensis* Pojark, and *C. dsungarica* Zabel.

Two populations of *S. laevigata* were found. The populations consisted mainly of adult, viable, fruit-bearing shrubs (70.3%), followed by young, fruit-bearing shrubs (24.8%). A small number of adult, diseased shrubs were also present (5%). The fruits were follicles with a dry appearance that split open to release the seeds. All plant accessions were moderately infested with *Aphis pomi*, and there was an incidence of powdery mildew and rust diseases. The surrounding plant species at the collection sites were *R. spinosissima*, *Daphne altaica* Pall., and *L. tatarica*.

Two populations of *S. persica* were identified. These populations consisted mainly of young (61.4%) and adult (38.6%) non-fruit-bearing trees of variable sizes ranging from 2 to over 4 m. Moderate rust damage and a complete absence of fruit were observed in both populations. According to the scientific staff of the Aksu Zhabagly State Nature Reserve, where population 1 was identified, this species has not produced fruit since 2021. The vegetation at the collection site consisted primarily of *Cerasus tianshanica* Pojark, *Crataegus turkestanica* Pojark, *Juniperus semiglobosa* Regel, *Filipendula* vulgaris Moench, and *Fraxinus sogdiana* Bunge.

Two populations of *S. schrenkianus* were identified. The populations consisted mainly of adult fruiting shrubs (95.8%) and a small number of young fruiting shrubs (4.2%). The plants varied in size, with most being less than 1 m in height (81.3%), followed by those between 1 and 1.7 m tall (14.6%), and a small proportion being greater than 1.7 m tall (4.2%). No disease incidence was observed, and the presence of spider cocoons (*Argiope bruennichi* Scopoli) was detected in only three accessions. The surrounding plant species at the collection sites included *Gaultheria procumbens* L., *R. platyacantha* Schrenk, *Eremurus tianschanicus* Pazij & Vved. ex Golosk., *Artemisia vulgaris* L., *Ephedra distachya* L. and, *Haloxylon persicum* Bunge ex Boiss. & Buhse.

Although the expeditions to different regions of Kazakhstan successfully identified a rich source of plant genetic diversity within the Rosaceae species, this valuable genetic pool is threatened by diseases, pests, aging of the plants, small population sizes, and climate change. The decline of these rare and endangered populations highlights the urgent need for ex situ conservation strategies to prevent the loss of these genetic resources [23,53,55,56].

### 2.2. In Vitro Establishment of Rosaceae Accessions

#### 2.2.1. Sexual Initiation Using Seeds or Embryonic Axes

**Figure 2:**
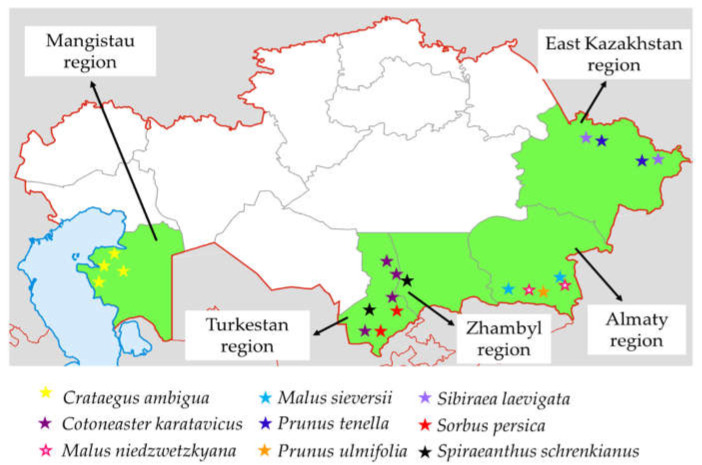
Map of regions in Kazakhstan where Rosaceae species were collected in 2023–2024. The collection sites are marked in green and the species are represented by colored stars.

In vitro techniques offer important options for the ex situ conservation of plant genetic resources. Seed banking, in particular, is the most efficient method for maintaining the genetic diversity [8,20,36,57]. The plant collection expedition successfully gathered fruits, and consequently, seeds, from eight accessions of Rosaceae species. Five of these accessions were established in vitro using seeds and/or embryonic axes isolated from the seeds (Table 1). The efficacy of in vitro initiation varied according to species. The high level of diversity among species, as well as their diverse natural habitats, may explain the observed responses [58,59,60]. In general, stratification at 4 °C increased the percentage of seed germination for all the species compared to the controls (C1–C3) (Table 1). For *C. ambigua*, seeds that were not stratified failed to germinate regardless of the method used. These seeds are protected by a hard sheath formed from the inner tissues of the pericarp [61,62,63]. In their natural habitat, seed germination is facilitated by the degradation of the pericarp, i.e., when the fruits fall to the ground, the pulp rots and the sheath softens, allowing the seeds to germinate more easily. Therefore, in this study, *C. ambigua* seeds were stratified and embryonic axes were isolated and used for in vitro initiation (Table 1, Figure 2). The same characteristics occurred with *C. karatavicus* seeds. Initial attempts at in vitro initiation using either the seeds or the isolated embryonic axes failed. For *C. ambigua,* the maximum percentage of viable shoots (52.6%) was obtained when the embryonic axes were surface sterilized in mercury chloride (HgCl_2_) for 4 min and grown on Knop medium. A slightly lower viability (42.2%) occurred when the embryonic axes were surface sterilized in HgCl_2_ for the same duration and grown on Murashige and Skoog medium (MS), though this difference was not significant [64]. Extending the sterilization time adversely affected the viability of the embryonic axes (Table 1). 

For *M. sieversii* and *S. laevigata*, the highest percentage of in vitro initiation was obtained from stratified seeds that were germinated ex vitro to provide shoots for initiation (E1). For *M. sieversii*, the highest shoot viability of 53.9% was obtained after 6 min sterilization in HgCl_2_. A slightly shorter HgCl_2_ treatment of 4 min resulted in the highest viability (61.5%) in *S. laevigata*. Interestingly, and in contrast to other species, *S. laevigata* exhibited high viability in controls where the seeds were not stratified, sterilized in HgCl_2_ for 4 min and were then germinated ex vitro to obtain shoots for induction (C1, 54.5%), as well as in seeds germinated in vitro on Knop medium (C2, 56.9%) (Table 1).

For *P. tenella*, the highest percentage of in vitro initiation (44.3%) was obtained from embryonic axes treated with HgCl_2_ for 7 min and germinated on Knop medium. For *S. schrenkianus*, seeds treated with HgCl_2_ for 5 min and plated on Knop medium produced the highest viability (E2/1, 41.1%). As observed for all species, seeds or embryos grown on MS medium had lower viability rates than those grown on Knop medium (Table 1). Although MS medium is commonly used for plant tissue culture [38,66], it is possible that, in some cases, nutrient saturation of the medium hinders shoot development in seeds and embryonic axes, as observed in this study. Previous studies on other plant species have shown that the salt type and concentration can affect seed germination and shoot development [36,67,68,69].

Sometimes, even apparently healthy-looking cultures may harbor endophytes, which are microorganisms living within plant tissues that are not eradicated after surface sterilization. Symptoms of infection in tissue cultures may not appear until after several rounds of subculturing and/or after the cultures have undergone stress [28,70,71,72]. To minimize the risk of endophyte contamination, we screened the healthy-looking shoots for the presence of endogenous contaminants using a specialized medium that promotes their proliferation. Using the 523 medium, we observed variable levels of endophytic contamination among the different species. On average, 84.8% of the healthy-looking shoots did not reveal the presence of endophytic contamination (Table 1). *S. laevigata* and *S. schrenckianus* exhibited no signs of infection. A low level (<30%) of infection was observed in *P. tenella* and *M. sieversii*. A high level (60%) of infection was observed in *C. ambigua* (Table 1). All shoots corresponding to the infected tissue were discarded as potentially contaminated. Clean cultures of five Rosaceae species were used for further in vitro propagation experiments.

#### 2.2.2. Asexual Initiation Using Shoots

In vitro tissue culture also provides important approaches for both clonal propagation and the short- and medium-term storage of plant genetic resources [20,28,31,36,37,38,56]. When clonal propagation/preservation is required, i.e., when an exact copy of the parent tree is needed, vegetative propagules, such as shoots, are preferable to seeds because they can maintain a high level of genetic fidelity [30,49]. In addition, clonal propagules can also preserve selected individuals within a population that display desirable traits, such as disease resistance [28]. During the expedition, shoots and cuttings (clonal propagules) were collected from actively growing and dormant mother plants, respectively. In vitro clonal initiation was performed using shoots from eight *M. sieversii* accessions and seven *P. ulmifolia* accessions were used as a source of plant material. 

For *M.* sieversii, shoots were taken either directly from field-grown plants or from dormant cuttings that were forced indoors, surface-sterilized with HgCl_2_ for 7 min, and then used for in vitro initiation. We found that all eight accessions could be effectively established in in vitro cultures using shoots from both sources. On average, 62% and 48% of cultures were successfully initiated using shoots from dormant cuttings forced indoors and shoots taken directly from the field, respectively. Overall, shoots from dormant cuttings forced indoors resulted in the highest percentage of in vitro establishment across all accessions (Table 2). We have previously optimized these initiation procedures, including the extent of sterilization with HgCl_2_ and successfully initiated in vitro cultures of *Malus* cultivars and rootstocks [26]. 

For *P. ulmifolia,* in vitro establishment was performed using only shoots collected from dormant cuttings that were forced indoors (Table 2). Six out of seven accessions were successfully established in vitro with variable efficiencies ranging from 22% to 100%. As was observed for *M. sieversii*, significant differences were observed among the accessions regarding the number of viable shoots following HgCl_2_ exposure. However, a higher degree of variability was observed for *P. ulmifolia* accessions. The results of the present study show that the previously optimized protocol for *Malus* cultivars and rootstocks can also be applied to the wild *Malus* and *Prunus* species.

As was previously carried out for asexual initiation, healthy-looking clonal shoots were also screened for the presence of endogenous contaminants. Indexing on 523 medium revealed significant differences in contamination levels between *M. sieversii* shoots from different sources. A significantly lower percentage of endophytic contamination was observed in shoots collected from dormant cuttings forced indoors than in shoots taken directly from the field. Aseptic shoot levels ranging from 37.5% to 63% were obtained across eight *M. sieversii* accessions. On average, 55.9% of in vitro shoots were free of contamination, compared to 30.5% of shoots taken directly from the field (Table 2). This finding is consistent with the results of previous studies [27,37,68,73,74] and is possibly due to a higher degree of contamination in shoots harvested from field collections compared to those sprouted in a more controlled environment. Additionally, the present protocol involved surface sterilizing of dormant cuttings prior to inoculation, which reduced the level of contamination. However, both sources of shoots remain relevant, offering the possibility of in vitro initiation at different times of the year and in situations where starting plant material is scarce [27]. Screening on 523 medium revealed that the aseptic shoot levels of *P. ulmifolia* ranged from 35.6% to 60% across seven accessions that were initiated from shoots collected from dormant cuttings that were forced indoors. On average, 51.3% of the in vitro shoots were free of contamination.

### 2.3. In Vitro Multiplication

Four-week-old cultures of *C. ambigua*, *P. tenella*, *S. laevigata*, and *S. schrenkianus*, established from sexual initiation and considered clean after screening on Media 523, were used for our in vitro propagation. We used a variety of MS-based media for the propagation experiments. Our input parameters included various phytohormones: 6-benzylaminopurine (BAP), indolylbutyric acid (IBA), and gibberellic acid (GA_3_). The concentrations were genotype-specific and based on previous studies conducted by our team. Shoot proliferation was achieved by promoting axillary shoot growth at a variable multiplication rate (MR) across the four Rosaceae species. The MR averaged 1.8 for *C. ambigua*, 2.3 for *P. tenella*, 4.2 for *S. laevigata*, and 1.9 for *S. schrenckianus*. We found that the concentration of phytohormones influenced both the MR and the quality of the in vitro cultures (Table 3).

For *C. ambigua*, the strength of the MS medium and phytohormone concentration were the most effective factors for shoot proliferation. Media nos. 9 and 10, which were based on 1/2 MS with a reduced amount of BAP (0.2 mg L^−1^), produced the highest MR, with 3.3 and 3.5 shoots per initial explant, respectively. Furthermore, media nos. 9 and 10 were supplemented with iron and vitamin C. This significantly increased the vigor and MR of the in vitro cultures compared to medium no. 8, which was also MS-based medium (Table 3). The positive effects of supplementing the culture media with iron and vitamin C on improved rooting, vigor, shoot proliferation, and overall in vitro plant growth are well documented [66,75,76,77,78,79]. Similar to *C. ambigua*, *P. tenella* in vitro cultures grown on 1/2 MS supplemented with iron and reduced levels of BAP (0.25 mg L^−1^, medium no. 11) produced the highest MR of 3.2 shoots per initial explant compared to the other 12 media formulations. Although not significant, there was a slight decrease observed in MR: 2.9 for medium no. 12 and 2.8 for medium no. 10, when cultures were grown on the same formulation as medium no. 11, except for lacking AIB or doubled BAP, respectively (Table 3). The combination of auxin and cytokinin in the culture media, especially at the optimum concentration, enhances in vitro plant growth and development. Higher levels of cytokinin together with lower levels of auxin (i.e., a high cytokinin/auxin ratio), are usually synergistically suitable for cell division and regeneration in vitro. The high cytokinin/auxin ratio has been shown to promote cell division, growth, and proliferation in vitro in a wide range of plant species [27,28,51,80,81,82], including other *Prunus* species [83,84]. Interestingly, *P. tenella* exhibits low reproductive rates and therefore slow population growth in its natural habitat, which may contribute to its current endangered status in Kazakhstan [85,86]. Additionally, other studies using plant tissue culture have found this species to be less prolific, corroborating our findings [87,88,89].

For *S. laevigata*, a higher ratio of 50 to 100:1 of auxin to cytokinin in the MS media, along with the addition of 0.2 mg L^−1^ GA_3_, resulted in the highest MR of 5.5, compared to the optimized media for the other species. The same number of shoots was obtained despite doubling the amount of BAP from 0.5 to 1 mg L^−1^. Therefore, it is recommended to use the MS medium supplemented with 0.5 mg L^−1^ BAP, 0.01 mg L^−1^ IBA, and 0.2 mg L^−1^ GA_3_ (medium no. 8) for this species (Table 3). Culture media supplemented with GA_3_ in combination with auxin and/or cytokinin can sometimes stimulate shoot proliferation [66,90,91,92,93], though it is more often used to promote shoot elongation [93,94]. The optimized medium for *S. laevigata* did not stimulate the proliferation of shoots in *S. schrenkianus*. MS medium supplemented with 0.1 mg L^−1^ BAP and 0.01 mg L^−1^ IBA (medium no. 7) resulted in the highest MR of 4.0 shoots per initial explant for *S. schrenkianus*. A slightly lower MR of 3.5 shoots, but not significantly different from medium no. 7, was obtained by increasing the concentrations of BAP and IBA to 0.2 and 0.02 mg L^−1^, respectively (medium no. 6). Although different concentrations of BAP and IBA were used in media nos. 6 and 7, both formulations have the same 10:1 cytokinin/auxin ratio (Table 3).

### 2.4. Seed Storage at −20 °C or Storage of Seeds and Embryonic Axes at Cryogenic Temperature

Seeds from different accessions of *M. niedzwetzkyana*, *M. sieversii*, *S. laevigata*, and *S. schrenckianus* were successfully stored at −20 °C or under cryopreservation conditions (−196 °C), and average viability across species was 86.4% and 94.0%, respectively (Table 4). These results demonstrate that the two methods are effectively comparable and offer different possibilities for genebanks. For genebanks without cryopreservation facilities, storing seed at −20 °C remains an effective method for the Rosaceae plant species tested. The seed storage procedures used in the current study were established through our previous work with various plant species. We have successfully stored and regenerated seeds of various genera, including *Malus*, *Berberis*, *Lonicera*, *Linum*, *Valeriana*, *Silybum*, and *Matricaria*, as well as some wild Poaceae species [57,95,96,97,98,99]. To the best of our knowledge, this study is the first to report on seed conservation efforts for *S. laevigata* and *S. schrenckianus*, two endangered and rare species listed in the Red Book of Kazakhstan.

In addition to seed banking, the use of embryonic axes as a source of propagules for cryopreservation has been studied for *M. sieversii*, *C. ambigua*, and *P. tenella*. Embryonic axes have been used as a source of explants in cryopreservation studies, such as for species that produce recalcitrant seeds, but they have not been used as extensively as seeds or shoot tips [52,53,56,100]. Embryonic axes are often cryopreserved using methods involving highly concentrated cryoprotectant solutions prior to exposure to LN [101,102,103,104]. These solutions osmotically remove most of the water from cells, preventing ice crystal formation and minimizing damage during freezing and thawing [31,44]. Alternatively, desiccation procedures can be achieved through physical dehydration by partially drying tolerant tissues prior to exposure to LN [31,32,33,34]. In this study, we applied a simplified, and user-friendly cryopreservation protocol that does not require cryoprotectants. This protocol was previously used by our team to conserve embryonic axes of *M. sieversii*, *Berberis* spp., and *Glycine max* [96,97]. The embryonic axes of the three species were dehydrated by air-drying in a laminar flow hood for 40 min to reach a moisture contents of 7.2% for *C. ambigua*, 6.9% for *M. sieversii*, and 10.1% for *P. tenella*, prior to being exposed to LN. The cryovials containing the embryonic axes were thawed at 4 °C for 1 h, after which the embryonic axes were plated on recovery medium. This procedure resulted in viability ranging from 60.0% to 73.3% for *C. ambigua*, 50.0% to 66.7% for *M. sieversii*, and 70.0% to 80.0% for *P. tenella*. On average, 65.7.9% of the embryonic axes were successfully recovered after cryopreservation across the three species (Table 4).

### 2.5. Creation of a Cryogenic DNA Bank

As an additional effort of this study, leaf samples were collected during the expeditions from the eight Rosaceae species (*C. ambigua*, *M. niedzwetzkyana*, *M. sieversii*, *P. tenella, P. ulmifolia, S. laevigata, S. persica,* and *S. schrenkianus*). To effectively preserve the quality of the genetic material for future use, DNA was extracted from the leaves and stored in LN. The amount of total DNA extracted varied depending on the plant species. It varied from 180 to 320 µg/µL for *C. ambigua*, 151 to 274 µg/µL for *M. niedzwetzkyana*, 137 to 225 µg/µL for *M. sieversii*, 203 to 289 µg/µL for *P. tenella*, 221 to 258 µg/µL for *P. ulmifolia*, 193 to 274 µg/µL for *S. laevigata*, 149 to 208 µg/µL for *S. persica*, and 199 to 257 µg/µL for *S. schrenkianus*. The quality of the isolated DNA was measured by A260/A280 ratio, which was within the range of 1.6 to 1.9. These results indicate the high quality of the isolated DNA and the low level of sample contamination. Once cryopreserved, samples preserved as DNA are relatively easy to handle and store, and most importantly, DNA integrity is maintained for future use [56].

## 3. Materials and Methods

### 3.1. Plant Material Acquisition

Rosaceae species were collected during expeditions conducted in 2023–2024 in 5 regions of Kazakhstan, namely Almaty, East Kazakhstan, Zhambyl, Mangistau and Turkestan. Figure 2 shows the locations where the plant material was collected. A total of 250 accessions representing 9 species, consisting of 35 *C. karatavicus*, 40 *C. ambigua*, 3 *M. niedzwetzkyana*, 55 *M. sieversii*, 21 *P. tenella*, 15 *P. ulmifolia*, 31 *S. laevigata*, 26 *S. persica*, and 20 *S. schrenkianus*, were collected during the expeditions. The accessions were characterized according to the descriptors established by the Food and Agriculture Organization of the United Nations [105], the International Plant Genetic Resources Institute, the European Cooperative Program on Plant Genetic Resources, and Lateur et al. [106]. The evaluation included 35–49 parameters, tailored to each species, as shown in Supplementary Table S1.

The selection of plant material was not intentional, but situational, i.e., plant material was collected from trees at the collection sites based on actual field encounters. Geographic coordinates were recorded using the eTREX^®^H Garmin Montana 750i GPS navigator along with observations of each accession collected in situ. The sizes (height or width) of the tree accessions were determined using a standard tape measure. The presence of diseases and pests was assessed visually. In addition, photographs were taken using a Canon EOS RP RF 24-105 F4-7.1 IS STM camera and an iPhone 14 Pro mobile phone for further evaluation. Within a 10 m radius of each accession, 5 to 10 species of surrounding plants were recorded. Each accession was labelled with a unique tag containing the collection number and date. Table 5 lists the collection sites, GPS coordinates and elevation of the exact collection point.

The collections included fruits, leaves, cuttings and shoots. During the fruiting season, which ranged from July to September, a sample of mature fruits was collected from each accession. The amount varied according to species and availability. The fruits were carefully placed in 250 mL plastic or polyethylene containers, transported to the laboratory, stored at room temperature, and used within 5 days of collection. Random samples of leaves (10–30 pieces) were collected from each accession from July to September. The leaves were placed between two layers of filter paper, placed in hermetically sealed bags containing 20 g of silica gel, and transported to the laboratory. The leaves were stored at room temperature and used for species characterization and DNA extraction. At the same time, a few 15–30 cm shoots were collected from each accession and placed between thick, folded sheets of cardboard, and used for herbarium preparation. Shoots were also harvested from April to June and used as a source of plant material for in vitro initiation. In February and March, one-year-old cuttings approximately 25–40 cm long with 7–10 buds, were harvested from dormant mother plants, transported to the laboratory, and used as a source of plant material for in vitro initiation.

### 3.2. In Vitro Establishment of Rosaceae Accessions

#### 3.2.1. Sexual Initiation Using Seeds or Embryonic Axes

Fruits were washed with soapy water and dried on paper towels. Seeds were de-pulped by hand and surface-sterilized by immersing them in 50% commercial bleach (Belizna^®^, 5–15% sodium hypochlorite, alkaline components ≤ 5%) for 5 min with occasional stirring and rinsed three times with sterile water.

For experiment 1 (E1), the seeds were placed in 300 mL plastic containers filled with perlite (Union Perlite, Almaty, Kazakhstan) and stratified at 4 °C for 8 weeks under an 8 h photoperiod with a light intensity of 10 µmol m^−2^ s^−1^. After stratification, the containers were transferred to a temperature of 24 ± 1 °C and a photoperiod of 16 h at a photosynthetic flux density of 40 µmol m^−2^ s^−1^ with two types of OPPLE tubular fluorescent lamps: YK 21RR 16/G 21W 6500K RGB and YK 21RL 16/G 21W 4000K RGB, supplied by ElectroComplex in Corporation (Almaty, Kazakhstan) (hereafter, called standard conditions). After 3–15 days, the resulting 1–2 cm shoots containing a single node were sterilized with a 0.1% HgCl_2_ solution for 4–7 min. Afterwards, shoots were placed in 150 mm × 25 mm test tubes of MS medium [64] containing 30 g L^−1^ sucrose, 0.5 mg L^−1^ BAP, 0.02 mg L^−^IBA, 1.75 g L^−1^ Gelrite™ (PhytoTechnology Laboratories^®^, Lenexa, KS, USA), and 4 g L^−1^ agar (PhytoTechnology Laboratories^®^, Lenexa, KS, USA) at pH of 5.7. Cultures were incubated in a growth room maintained under standard conditions.

For experiment 2, seeds were stratified as described above and then the whole seed or the isolated embryonic axes (*M. sieversii*—0.95 mm; *P. tenella* —1.7 mm) were sterilized in 0.1% HgCl_2_ solution for 4–9 min. Sterilized seeds were sown in test tubes (1 seed per test tube) containing Knop medium [107] (E2/1) or MS medium (E2/3) solidified with 1.75 g L^−1^ Gelrite™, 4 g L^−1^ agar at pH 5.7. Sterilized embryonic axes were sown in test tubes (1 seed per test tube) containing Knop medium (E2/2) or MS medium (E2/4) solidified 1.75 g L^−1^ Gelrite™, 4 g L^−1^ agar at pH 5.7. Cultures were incubated in a growth room maintained under standard conditions.

As controls, seeds without stratification were germinated in perlite and shoots were sterilized in 0.1% HgCl_2_ for 4–9 min (C1) as described above, or seeds sterilized in 0.1% HgCl_2_ for 4–9 min were germinated in test tubes containing Knop medium (C2) or MS medium (C3) supplemented with 1.75 g L^−1^ Gelrite™, 4 g L^−1^ agar at pH 5.7. Cultures were incubated in a growth room maintained under standard conditions.

#### 3.2.2. Asexual Initiation Using Shoots

Two techniques were used for clonal initiation using shoots, following the procedure previously published by our team [27,28]. Cultures of eight *M. sieversii* accessions were initiated with young shoots taken directly from the trees at the collection site from late April to June. Alternatively, shoots were sprouted in an indoor laboratory environment from cuttings taken from dormant mother plants of the eight *M. sieversii* accessions and seven *P. ulmifolia* accessions.

First technique: shoots of 2–3 cm with a single bud, harvested directly from the field, were washed with soapy water and treated with a 0.1% HgCl_2_ solution for 7 min.

Second technique: dormant cuttings were washed with soapy water, rinsed and treated with 50% commercial bleach (Belizna^®^, 5–15% sodium hypochlorite, alkaline components ≤ 5%) for 5 min and rinsed in running water. Cuttings were immediately placed in a container with water where the base of the cutting was submerged in water and kept under laboratory conditions (24 ± 1 °C and a photoperiod of 10 h 30 min) for bud sprouting. The water at the base of the cuttings was replaced daily. After 2–4 weeks, shoots that had grown to 1–2 cm and contained a single bud were cut and washed in soap water, sterilized in 0.1% HgCl_2_ for 7 min.

**Figure 3:**
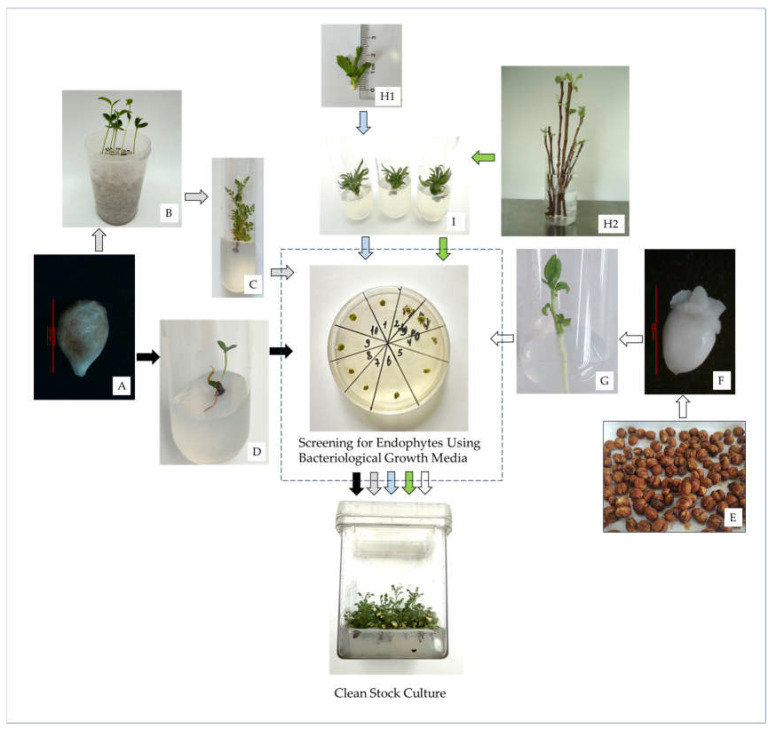
Flowchart depicting the strategies used for the in vitro establishment of Rosaceae species using sexual and asexual sources of explants. (**A**) Seeds were either placed in (**B**) plastic containers filled with perlite and stratified at 4 °C for 8 weeks before being exposed to 24 ± 1 °C and germinated to provide shoots for (**C**) in vitro initiation, or stratified at 4 °C for 8 weeks, sterilized in mercuric chloride (HgCl_2_) and (**D**) germinated on culture media. As an alternative strategy, (**E**) seeds were stratified at 4 °C for 8 weeks, (**F**) embryonic axes were isolated, sterilized in HgCl_2_ and (**G**) germinated on culture media. Shoots were taken either directly from the (**H1**) plants in the field or by (**H2**) sprouting new shoots (in the laboratory) from cuttings taken from dormant mother plants and used as explants for (**I**) in vitro initiation. In vitro cultures were then screened for endophytes using bacteriological growth media and healthy-looking shoots were used to establish stock cultures.

For both initiation techniques, surface sterilized shoots were then placed in test tubes with MS medium supplemented with 30 g L^−1^ sucrose, 0.5 mg L^−1^ BAP, 0.02 mg L^−1^ IBA, 1.75 g L^−1^ Gelrite™ (PhytoTechnology Laboratories^®^, Lenexa, KS, USA), and 4 g L^−1^ agar (PhytoTechnology Laboratories^®^, Lenexa, KS, USA) at pH of 5.7. Cultures were incubated in a growth room maintained under standard conditions. Figure 3 shows a flowchart depicting the strategies used for in vitro establishment of Rosaceae species using sexual and asexual sources of explants.

#### 3.2.3. Screening for Endophytes Using Bacteriological Growth Media

After four weeks of in vitro initiation, the basal parts of healthy-looking in vitro shoots (3–5 mm) derived from seeds and embryonic axes germinated in vitro, and shoots harvested from sprouted cuttings were placed in Petri dishes (100 mm × 15 mm) containing bacteriological growth medium 523 [65]. Medium 523, a general medium for growth of bacteria is composed of 10 g L^−1^ sucrose, 8 g L^−1^ casein hydrolysate (Sigma-Aldrich, St. Louis, MO, USA), 4 g L^−1^ yeast extract, 2 g L^−1^ monopotassium phosphate (KH_2_PO_4_), 0.15 g L^−1^ magnesium sulfate (MgSO_4_·7H_2_O), and 6 g L^−1^ Gelrite™ at pH 6.9 [65]. Plates were incubated at 24 ± 1 °C for 2 weeks to promote endogenous pathogen proliferation. Therefore, cloudiness of the media or growth of colonies on medium 523 indicates contamination. The percentages of clean and contaminated shoots were recorded, and the healthy-looking shoots were used for further propagation experiments [70].

### 3.3. In Vitro Multiplication

Different MS-based formulations supplemented with genotype-specific phytohormone concentrations (Table 6) were used to optimize the culture medium during the in vitro multiplication phase. Cultures of *C. ambigua*, *P. tenella*, *S. laevigata*, and *S. schrenkianus* established in vitro from sexual initiation were used as the source of explants to determine the multiplication rate (MR). Nodal sections (1–2 cm and a single bud) from 4 Rosaceae species) were taken from 4-week-old in vitro plants and placed in 237 mL glass culture vessels (PhytoTechnology Laboratories^®^, Lenexa, KS, USA) at a density of 5 nodal sections per vessel. Cultures were incubated in a growth room maintained under standard conditions.

The MR was calculated in shoots growing for 5 weeks on one of the culture media shown in Table 3. Cultures were subcultured twice every 5 weeks on the same medium formulation. MR was calculated using the formula: MR = a/bc, where a is the number of new shoots, b is the number of initial shoots, and c is the number of subcultures.

### 3.4. Seed Storage at −20 °C or at Cryogenic Temperatures

**Figure 4:**
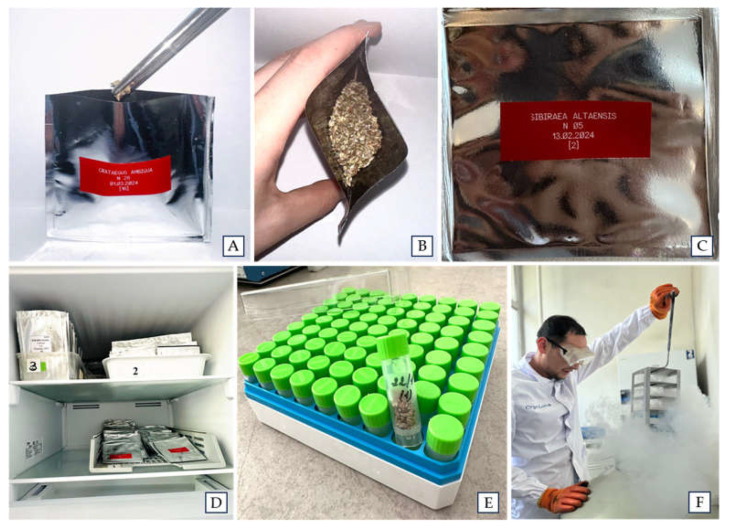
Seeds of Rosaceae species stored at the Institute of Plant Biology and Biotechnology, Almaty, Kazakhstan. Seeds were partially dehydrated and transferred to 90 × 90 mm aluminum foil laminate bags (**A**,**B**), hermetically sealed (**C**) and stored at −20 °C (**D**), or transferred to cryovials (**E**) and stored at −196 °C (**F**).

Depending on availability, 10–50 seeds of *M. niedzwetzkyana* and *M. sieversii*, as well as 100 seeds of *S. laevigata* and *S. schrenckianus*, were air-dried at 24 ± 1 °C and 20–22% relative humidity for 1 week without pre-treatment. After dehydration, the moisture content of the seeds varied from 7.9% to 11.2%, with an average of 9.2%. Seed loot of 10–100 seeds were then hermetically sealed in 90 × 90 mm aluminum foil laminate bags and stored at −20 °C (freezer BEKO B1RFNK312W, Technodom LLC) (Figure 4).

*M. niedzwetzkyana* and *M. sieversii* seeds (10 pcs.) and *S. laevigata* and *S. schrenckianus* (100 pcs.) were also stored at cryogenic temperatures (−196 °C). Seeds were dehydrated as described above, placed in 1.2 mL cryovials (Corning^®^, Corning, NY, USA) containing 5–100 seeds per vial, and stored in LN (Figure 3).

After 3 days of storage at −20 °C or 1 h in LN, the aluminum bags or cryovials containing seeds were thawed at 24 ± 1 °C for 30 min in a laminar flow hood. Seeds were then transferred to 150 mm × 25 mm test tubes (one seed per test tube) on hormone-free Knop medium [107] jellified with 1.75 g L^−1^ Gelrite™, 4 g L^−1^ agar at pH 5.7. Seeds were incubated in a growth chamber maintained under standard conditions, and seed viability was assessed after 2 weeks. Seeds were considered viable when the radicle emerged (>5 mm) and the shoots developed into a full plantlet.

### 3.5. Creation of a Cryogenic Embryonic Axes Bank

Embryonic axes of *C. ambigua* (1.2 mm), *M. sieversii* (1.1 mm), and *P. tenella* (2 mm) were sterilized in a 0.1% HgCl_2_ solution for 5 min and placed in an empty sterile Petri dish (100 mm × 15 mm; 10 embryonic axes per plate). The Petri dish containing the embryonic axes were then dehydrated by airflow in a laminar flow hood for 40 min at 24 ± 1 °C and 20–22% relative humidity [96]. After dehydration, the water content of the embryonic axes was determined using a humidity analyzer (KERN MLB 50-3, Balingen, Germany). Embryonic axes dehydrated to a moisture content of 7.2% for *C. ambigua*, 6.9% for *M. sieversii* and 10.1% for *P. tenella* were transferred to 1.2 mL cryovials (5–10 embryos per cryovial) and immersed in LN. After 1 h in LN, the cryovials containing the embryonic axes were thawed at 4 °C for 1 h and then the embryonic axes were plated in 150 mm × 25 mm test tubes with MS medium supplemented with 30 g L^−1^, 1.75 g L^−1^ Gelrite™, 4 g L^−1^ agar at pH 5.7. Embryonic axes were incubated in a growth chamber maintained under standard conditions and viability was assessed after 1–2 weeks. Embryonic axes were considered viable when the radicle emerged (> 5 mm) and the shoots developed into a full plantlet.

### 3.6. Creation of a Cryogenic DNA Bank

To effectively preserve the quality of genetic material for decades for research or other purposes, DNA was extracted from leaves and then stored in LN. Leaves from *C. ambigua, M. niedzwetzkyana, M. sieversii, P. tenella, P. ulmifolia, S. laevigata, S. persica,* and *S. schrenkianus* were used to extract DNA using the method described by Doyle and Doyle [108]. The Doyle and Doyle extraction buffer consists of 100 mM Tris-HCl (pH 8.0), 20 mM EDTA, 1.4 M NaCl, 2% (*w*/*v*) CTAB, 2% (*w*/*v*) polyvinylpyrrolidone (PVP) and 0.2% (*w*/*v*) 2-β-mercaptoethanol. Plant tissue (200 mg) was pulverized with 1 mL of extraction buffer using a high-speed, low-temperature tissue homogenizer KZ-III-F (Wuhan Servicebio Technology Co., Ltd., China) with steel beads (3 mm). Homogenization parameters were as follows 4 °C, 70 Hz, run time—60 s, pause time—20 s 5 times. The quality and quantity were evaluated using a nanodrop spectrophotometer (Eppendorf BioSpectrometer^®^ basic SN 6135JP304854). After isolation, total DNA was diluted to a concentration of 100 µg/µL, each sample was placed in triplicate in cryogenic tubes and stored in LN for long-term storage. In addition, the integrity and quality of the isolated DNA was assessed by electrophoresis in 1.2% agarose gel dissolved in TBE buffer (Tris-borate-EDTA) and stained with ethidium bromide.

### 3.7. Statistical Analysis

For in vitro initiation experiments, a minimum of 20 shoots were used per replicate with three replicates per treatment in a randomized block design. The total number of shoots indexed using bacterial nutrient medium 523 varied from 5 to 30, depending on the availability of accessions obtained in each accession after initiation. The in vitro multiplication experiment was performed with two replicates of 15 shoots for each accession. For the cryopreservation experiments, 5–10 seeds or embryonic axes, depending on availability, were used per replicate with three replicates. As a control for the cryopreservation experiments, the viability of dehydrated seeds and embryo axes was accessed by planting them in the recovery medium without immersion in LN. Means and standard error (SE) were calculated across experimental replicates and analyzed using analysis of variance, and Tukey’s mean separation test was performed. *p* ≤ 0.05 was considered significantly different. All analyses were performed using SYSTAT 12.0 software package [109].

## 4. Conclusions

This study focused on geobotanical and ex situ conservation efforts for rare and endangered Rosaceae species listed in the Red Book of Kazakhstan. The loss of unique endemic plants, such as those in this study, can lead to significant ecological damage and potentially irreversible consequences. The geobotanical study revealed an alarming trend of biodiversity loss driven by disease and pest threats, which were frequently observed in different populations/regions. Other factors contributing to this loss include the aging of the plants, the small population sizes, and the weak in situ fruiting. This study successfully contributes to the conservation of Rosaceae species by employing various propagation and conservation strategies. These strategies include sexual and asexual propagation, as well as conserving plant material in vitro or at low temperatures. This involved ex situ conservation using different propagules (e.g., seeds, embryonic axes, and shoots) and storage methods (e.g., in vitro culture, seed banking, and cryopreservation) for the same species. This enables future generations to use this valuable reservoir of genetic diversity for crop improvement and may serve as a basis for propagating planting material to restore degraded populations.

## Figures and Tables

**Table 1:** Effects of different durations of surface sterilization with mercuric chloride (HgCl_2_) on the in vitro establishment of Rosaceae species using seeds, embryonic axes or shoots taken from germinated seeds as explants and percentage of clean shoots indexed using the bacterial growth medium 523.

Experimental Variant	*N*	HgCl_2_ Exposure Duration (min)					Indexing with Medium 523	
		4	5	6	7	9		
		Viability of In Vitro Shoots (%)					*N*	Clean Shoots (%)
*Crataegus ambigua*								
E1	60	-	0 e	-	0 e	-	-	-
E2/2	80	52.6 ± 8 ab	-	7.3 ± 4 cd	-	0 e	15	40.3 ± 7 cd
E2/4	80	42.2 ± 5 b	-	31.7 ± 7 bc	-	7.7 ± 5 cd	9	39.8 ± 5 cd
C1	80	0 e	-	0 e	-	0 e	0	-
*Malus sieversii*								
E1	60	29.4 ± 5 bc	-	53.9 ± 4 ab	-	-	16	72.8 ± 5 b
E2/1	60	23.7 ± 12 bc	-	37.1 ± 4 b	-	-	14	75.1 ± 14 b
E2/3	60	15.6 ± 12 c	-	19.1 ± 5 c	-	-	6	85.0 ± 6 b
C1	60	17.8 ± 13 c	-	22.7 ± 12 bc	-	-	6	66.7 ± 8 c
C2	60	12.8 ± 14 cd	-	17.6 ± 12 c	-	-	11	72.7 ± 7 b
*Prunus tenella*								
E2/2	80	22.5 ± 11 bc	-	-	44.3 ± 7 b	0 e	12	71.4 ± 9 b
E2/4	80	14.9 ± 13 cd	-	-	19.5 ± 13 c	0 e	5	72.8 ± 9 b
C1	60	0 e	-	-	0 e	-	-	-
*Sibiraea laevigata*								
E1	60	61.5 ± 12 a	-	38.2 ± 12 b	-	-	30	100.0 ± 0 a
E2/1	60	56.2 ± 6 ab	-	36.7 ± 13 b	-	-	29	100.0 ± 0 a
E2/3	60	33.4 ± 14 bc	-	25.8 ± 12 bc	-	-	18	100.0 ± 0 a
C1	60	54.5 ± 13 ab	-	34.0 ± 13 bc	-	-	27	100.0 ± 0 a
C2	60	56.9 ± 12 ab	-	37.3 ± 6 b	-	-	28	100.0 ± 0 a
C3	60	27.1 ± 14 bc	-	26.9 ± 5 bc	-	-	16	100.0 ± 0 a
*Spiraeanthus schrenckianus*								
E1	60	-	27.3 ± 12 cd	-	26.9 ± 12 bc	-	19	100.0 ± 0 a
E2/1	60	-	41.1 ± 12 b	-	35.6 ± 14 bc	-	23	100.0 ± 0 a
E2/3	60	-	20.9 ± 15 c	-	12.9 ± 12 cd	-	10	100.0 ± 0 a
C1	60	-	0 e	-	0 e	-	-	-
C2	60	-	30.2 ± 21 bc	-	20.5 ± 12 c	-	15	100.0 ± 0 a
C3	60	-	13.7 ± 12 cd	-	9.5 ± 12 cd	-	14	100.0 ± 0 a
**Mean**		30.6 ± 18	20.5 ± 15	29.3 ± 13	16.9 ± 13	1.5 ± 3.4	14	84.8 ± 33

Data represent mean ± SE. Values accompanied by different letters in each section were significantly different at *p* ≤ 0.05 using Tukey’s mean separation test. Medium 523 is a general medium for the growth of bacteria [65]. *N*, number of shoots used for each treatment; HgCl_2_, mercuric chloride. E1—Seeds were placed in plastic containers filled with perlite and stratified at 4 °C for 8 weeks before being exposed to 24 ± 1 °C and germinated to provide shoots for initiation. E2/1—seeds were stratified at 4 °C for 8 weeks, sterilized in HgCl_2_ and germinated on Knop medium or MS medium (E2/3); E2/2—seeds were stratified at 4 °C for 8 weeks, embryonic axes were isolated, sterilized in HgCl_2_ and germinated on Knop medium or MS medium (E2/4); C1—seeds without stratification were germinated in perlite and shoots were sterilized in HgCl_2_; C2—Seeds sterilized in HgCl_2_ were germinated in test tubes containing Knop or MS medium (C3).

**Table 2:** In vitro establishment (%) of shoots taken directly from the field of eight accessions of *Malus sieversii*, or by sprouting new shoots from cuttings taken from dormant mother plants of eight accessions of *Malus sieversii* and seven accessions of *Prunus ulmifolia* and sprouted in an indoor laboratory environment and surface sterilized with mercuric chloride (HgCl_2_) for 7 min; and percentage of clean shoots indexed using the bacterial growth medium 523.

Plant Species, Accession	In Vitro Establishment of Shoots								Indexing with Medium 523						
									Clean Shoots (%)						
	Sprouted Cuttings in the Laboratory (E1)		*N*		Taken Directly from the Field (E2)		*N*		Sprouted Cuttings in the Laboratory (E1)		*N*		Taken Directly From the Field (E2)		*N*
*M. sieversii*, 1	62 ± 13	b	39		30 ± 10	c	60		37.5 ± 8	b	24		31.1 ± 10	b	18
*M. sieversii*, 2	51 ± 6	b	40		40 ± 9	bc	60		59.2 ± 14	ab	18		30.6 ± 6	bc	24
*M. sieversii*, 3	48 ± 8	bc	32		34 ± 8	c	60		55.0 ± 18	ab	20		13.0 ± 12	c	20
*M. sieversii*, 4	63 ± 10	b	26		52 ± 5	b	60		56.2 ± 19	ab	16		31.7 ± 8	b	31
*M. sieversii*, 5	55 ± 13	b	28		56 ± 17	b	60		54.5 ± 18	ab	22		39.2 ± 14	b	23
*M. sieversii*, 6	79 ± 10	ab	32		51 ± 13	b	60		62.2 ± 17	a	15		15.0 ± 9	c	30
*M. sieversii*, 7	73 ± 8	ab	48		62 ± 12	b	60		63.0 ± 12	a	18		35.1 ± 13	b	37
*M. sieversii*, 8	65 ± 8	b	33		55 ± 18	b	60		59.4 ± 9	ab	23		48.2 ± 13	ab	33
Mean	62 ± 11				48 ± 11				55.9 ± 13				30.5 ± 11		
*P. ulmifolia*, 1	45 ± 14	bc	29		-	-	-		52.2 ± 14	ab	13		-	-	-
*P. ulmifolia*, 2	22 ± 8	c	37		-	-	-		47.8 ± 10	ab	8		-	-	-
*P. ulmifolia*, 3	25 ± 9	c	56		-	-	-		57.8 ± 10	ab	14		-	-	-
*P. ulmifolia*, 4	75 ± 14	ab	36		-	-	-		54.5 ± 19	ab	20		-	-	-
*P. ulmifolia*, 5	0 ± 0	d	28		-	-	-		-	-	-		-	-	-
*P. ulmifolia*, 6	100 ± 0	a	20		-	-	-		35.6 ± 10	b	19		-	-	-
*P. ulmifolia*, 7	94 ± 5	a	22		-	-	-		60.0 ± 10	a	18		-	-	-
Mean	52 ± 39								51.3 ± 12						

Data represent mean ± SE. Values accompanied by different letters in each section were significantly different at *p* ≤ 0.05 using Tukey’s mean separation test. Medium 523 is a general medium for the growth of bacteria [65]. *N*, number of shoots used for each treatment.

**Table 3:** Effect of the composition of the nutrient medium on the multiplication rate (MR) of in vitro Rosaceae species.

Culture Media	*Crataegus ambigua*			*Prunus tenella*			*Sibiraea laevigata*			*Spiraeanthus schrenckianus*		
	N of Shoots		MR	N of Shoots		MR	N of Shoots		MR	N of Shoots		MR
	Int	Reg		Int	Reg		Int	Reg		Int	Reg	
1	15	18	1.2 ± 0.1 e	15	42	2.8 ± 0.3 cd	15	39	2.6 ± 0.5 cd	15	18	1.2 ± 0.4 e
2	15	30	1.8 ± 0.2 de	15	30	2.0 ± 0.3 d	15	45	3.0 ± 0.4 c	15	15	1.0 ± 0.1 e
3	15	15	1.0 ± 0.1 e	15	37	2.5 ± 0.4 cd	15	66	4.4 ± 0.9 b	15	16	1.3 ± 0.3 e
4	15	18	1.2 ± 0.3 e	15	23	1.5 ± 0.2 de	15	83	5.5 ± 1.6 a	15	15	1.0 ± 0.1 e
5	15	17	1.1 ± 0.1 e	15	27	1.8 ± 0.3 de	15	39	2.6 ± 0.5 cd	15	18	1.2 ± 0.4 e
6	15	18	1.2 ± 0.3 e	15	26	1.7 ± 0.2 de	15	75	5.0 ± 1.5 ab	15	52	3.5 ± 0.7 b
7	15	15	1.0 ± 0.1 e	15	27	1.8 ± 0.2 de	15	69	4.6 ± 1.1 ab	15	60	4.0 ± 0.9 b
8	15	38	2.7 ± 0.4 cd	15	36	2.4 ± 0.3 cd	15	82	5.5 ± 1.4 a		-	-
9	15	50	3.3 ± 0.5 b	15	39	2.6 ± 0.5 cd		-	-		-	-
10	15	53	3.5 ± 0.7 b	15	44	2.9 ± 0.6 cd		-	-		-	-
11	-	-	-	15	48	3.2 ± 0.8 bc		-	-		-	-
12	-	-	-	15	42	2.8 ± 0.3 cd		-	-		-	-
13	-	-	-	15	33	2.2 ± 0.2 d		-	-		-	-
**Mean**	15	28	1.8 ± 1.0	15	35	2.3 ± 0.8	15	62	4.2 ± 1.2	15		1.9 ± 1.3

Data represent mean ± SE. Values followed by different letters were significantly different at *p* ≤ 0.05 using Tukey’s mean separation test. Int, initial; N, number; MR, multiplication rate; Reg, regenerated.

**Table 4:** Storage of Rosaceae seeds at −20 °C or seeds and embryonic axes under cryopreservation conditions (−196 °C).

Plant Species, Accession	Seed Storage at −20 °C			Seed Cryopreservation			Embryonic Axes Cryopreservation		
	Viability (%)		*N*	Viability (%)		*N*	Viability (%)		*N*
*C. ambigua*, 1	-	-	-	-	-	-	73.3 ± 7.3	ab	30
*C. ambigua*, 2	-	-	-	-	-	-	60.0 ± 10.0	bc	30
*M. niedzwetzkyana*, 1	93.3 ± 5.8	a	30	100.0 ± 0.0	a	30	-	-	-
*M. niedzwetzkyana*, 2	96.7 ± 5.4	a	30	93.3 ± 6.2	a	30	-	-	-
*M. sieversii*, 1	96.7 ± 6.1	a	30	100.0 ± 0.0	a	30	50.0 ± 10.0	bc	30
*M. sieversii*, 2	96.7 ± 5.8	a	30	93.3 ± 6.8	a	30		-	-
*M. sieversii*, 3	-	-	-	86.7 ± 7.6	ab	30		-	-
*M. sieversii*, 4	-	-	-	93.3 ± 5.8	a	30	60.0 ± 17.3	bc	30
*M. sieversii*, 5	-	-	-		-	-	66.7 ± 15.3	bc	30
*P. tenella*, 1	-	-	-	-	-	-	80.0 ± 9.2	ab	30
*P. tenella*, 2	-	-	-	-	-	-	70.0 ± 10.0	b	30
*S. laevigata*, 1	90.0 ± 7.3	ab	30	93.3 ± 5.8	a	30	-	-	-
*S. laevigata*, 2	100.0 ± 0.0	a	30	100.0 ± 0.0	a	30	-	-	-
*S. laevigata*, 3	83.3 ± 11.6	ab	30	100.0 ± 0.0	a	30	-	-	-
*S. laevigata*, 4	93.3 ± 5.8	a	30	100.0 ± 0.0	a	30	-	-	-
*S. laevigata*, 5	80.0 ± 17.3	ab	30	90.0 ± 7.3	ab	30	-	-	-
*S. schrenckianus*, 1	66.7 ± 12.4	bc	30	93.3 ± 5.8	a	30	-	-	-
*S. schrenckianus*, 2	50.0 ± 8.2	c	30	73.3 ± 11.6	b	30	-	-	-
*S. schrenckianus*, 3	90.0 ± 6.6	ab	30	100.0 ± 0.0	a	30	-	-	-
**Mean**	86.4 ± 12.7			94.0 ± 7.4			65.7 ± 9.9		

Data represent mean ± SE. Values followed by different letters were significantly different at *p* ≤ 0.05 using Tukey’s mean separation test. *N*, number of explants used for each treatment.

**Table 5:** The collection sites, GPS coordinates, and elevations from which the Rosaceae accessions were collected during the expeditions in the years 2023–2024.

Species	Number of Accessions	GPS Coordinates, Elevations, m	Place of Collection, year
*Crataegus ambigua*	10	N44°27′.42.2″–N44°27′45.8″ E50°35′. 22.2″–E50°35′ 25.1″, 51–126 m	P1 *—Mangistau region, Kizim tract, 2023
	10	N44°28′.26.3″–N44°28′.27.5″ E51°00′. 45.4″–E51°00′. 50.1″, 58–81 m	P2—Mangistau region, gorge. “Sultan Uly”, 2023
	10	N43°39′.02.7″–N43°39′.55.8″ E50°35′. 22.2″–E50°35′. 25.1″, 4–12 m	P3—Mangistau region, Mangyshlak experimental botanical garden, 2023
	10	N44°12′.53.8″–N44°12′.55.5″ E51°59′. 29.1″–E51°59′. 30.8″, 252–277 m	P4—Mangistau region, Western Karatau, Samal gorge, 2023
*Cotoneaster karatavicus*	21	N42°36′.064″–N42°40′.044″ E070°13′.444″–E070°23′.508″, 837–844 m	P1—Turkestan region, Sairam-Ugam national park, Kokbulak gorge, 2024
	9	N42°36′.123″–N42°36′.574″ E070°23′.671″–E070°24′.047″, 932–956 m	P2—Turkestan region, Tyulkubas district, Kulan reservoir, 2024
	9	N43°05′.101″–N43°05′.146″ E069°55′.351″–E069°55′.388″, 715–716 m	3—Turkestan region, Bayzhansai gorge, Ulken Buken river, 2024
*Malus niedzwetzkyana na*	2	N43°17′.896″–N43°17′.880″ E079°30′.902″–E079°30′.912″, 1646–1664 m	P1—Almaty region, Uyghur district, gorge. “Bol’shoy Kyrgyzsai”, 2024
	1	N43°40′.1080″, E076°59′.8702″, 600 m	P2—Almaty region, Ili district, 2024
*M. sieversii*	35	N43°17′.118″–N43°23′.036″ E77°34′.394″–E77°39′.850″, 819–1343 m	P1—Enbekshikazakh district, Almaty region, Turgen village, 2023
	19	N43°17′.938″– N43°20′.749″ E079°29′.802″–E079°37′.927″, 1585–1657 m	P2—Almaty region, Uyghur district, gorge. “Kyrgyzsai”, 2023–2024
	1	N43°38′.240″, E080°03′.255″, 745 m	P3—Almaty region, Uyghur district, Chundzha-Kolzhat highway, 2024
*Prunus tenella*	20	N49°42′.460″–N49°42′.466″ E083°43′.463″–E083°43′.492″, 710–710 m	P1—East Kazakhstan region, western Altai, vicinity of Pribrezhny vil., 2023
	1	N50°32′.305″, E083°54′.190″, 512 m	P2—East Kazakhstan region, outskirts of Ridder, 2024
*P. ulmifolia*	15	N43°13′29.106″, E76°54′51.132″, 867–880 m	Almaty region, Main Botanical Garden, 2023–2024
*Sibiraea laevigata*	10	N50°19′.254″–N50°19′.290″ E83°32′.514″–E083°33′.083″, 730–793 m	P1—East Kazakhstan region, Ridder city, Bystrukha river valley, 2023
	21	N50°17′.826″–N50°17′.855″ E083°29′.310″–E083°29′.379″, 873–880 m	P2—East Kazakhstan region, Ridder city, Gromotukha river floodplain, 2024
*Sorbus persica*	21	N42°24′.423″–N42°53′.915″ E70°34′.813″–E70°34′.874″,1752–1883 m	P1—–Turkestan region, Aksu Zhabagly State Nature Reserve, Kshi-Kaindy gorge, 2023
	5	N42°53′.910″–N42°53′.915″ E70°00′.086″–E70°00′.091″, 1044–1052 m	P2—Turkestan region, gorge “Aule Karabastau”, 2023
*Spiraeanthus schrenkianus us*	12	N42°44′35.33″–N42°44′39.81″ E71°00′01.53′’–E71°00′20.97′’, 1055–1079 m	P1—Zhambyl region, Zhualynsky district, Kuyuk pass, 2023
	8	N42°58′.608″–N42°58′.623″ E69°40′.705″–E69°40′.707″, 527–538 m	P2—Turkestan region, Karatau foothills, Ak-Mechet cave, 2023

* P1–P4—Number of populations. M, meters. The distances between populations were at least 50 km.

**Table 6:** Variants of MS-based formulation media used for in vitro propagation of Rosaceae species.

**Accession**	Variants of Media *
*Crataegus ambigua*	(1) MS with 30 g L^−1^ sucrose, 0.5 mg L^−1^ BAP, 0.01 mg L^−1^ IBA (2) MS with 30 g L^−1^ sucrose, 0.2 mg L^−1^ BAP, 0.01 mg L^−1^ IBA (3) MS with 30 g L^−1^ sucrose, 0.5 mg L^−1^ BAP, 0.1 mg L^−1^ IBA (4) MS with 30 g L^−1^ sucrose, 0.5 mg L^−1^ BAP, 0.02 mg L^−1^ IBA (5) MS with 30 g L^−1^ sucrose, 0.5 mg L^−1^ BAP, 0.03 mg L^−1^ IBA (6) MS with 30 g L^−1^ sucrose, 0.1 mg L^−1^ BAP, 0.01 mg L^−1^ IBA (7) MS with 30 g L^−1^ sucrose, 0.1 mg L^−1^ BAP, 0.1 mg L^−1^ IBA (8) ½ MS with 30 g L^−1^ sucrose, 0.2 mg L^−1^ BAP, 0.01 mg L^−1^ IBA (9) ½ MS with 30 g L^−1^ sucrose, 0.2 mg L^−1^ BAP, 0.01 mg L^−1^ IBA, 0.1 g L^−1^ Sequestrene 138 Fe (10) ½ MS with 30 g L^−1^ sucrose, 0.2 mg L^−1^ BAP, 0.01 mg L^−1^ IBA, 0.1 g L^−1^ Sequestrene 138 Fe, 1.0 mg L^−1^ Vitamin C
*Prunus tenella*	(1) MS with 30 g L^−1^ sucrose, 0.5 mg L^−1^ BAP, 0.01 mg L^−1^ IBA, 7.0 mg L^−1^ FeSO_4_·7H_2_O, 9.3 mg L^−1^ Na_2_EDTA 2H_2_O, 166 mg L^−1^ CaCl_2_ (2) ½ MS with 30 g L^−1^ sucrose, 0.5 mg L^−1^ BAP, 0.01 mg L^−1^ IBA, 7.0 mg L^−1^ FeSO_4_·7H_2_O, 9.3 mg L^−1^ Na_2_EDTA 2H_2_O, 166 mg L^−1^ CaCl (3) ½ MS with 20 g L^−1^ sucrose, 0.5 mg L^−1^ BAP, 0.01 mg L^−1^ IBA, 7.0 mg L^−1^ FeSO_4_·7H_2_O, 9.3 mg L^−1^ Na_2_EDTA 2H_2_O, 166 mg L^−1^ CaCl_2_; (4) ½ MS with 20 g L^−1^ sucrose, 1.0 mg L^−1^ BAP, 0.01 mg L^−1^ IBA, 7.0 mg L^−1^ FeSO_4_·7H_2_O, 9.3 mg L^−1^ Na_2_EDTA 2H_2_O, 166 mg L^−1^ CaCl_2_ (5) ½ MS with 20 g L^−1^ sucrose, 1.0 mg L^−1^ BAP, 0.05 mg L^−1^ IBA, 7.0 mg L^−1^ FeSO_4_·7H_2_O, 9.3 mg L^−1^ Na_2_EDTA 2H_2_O, 166 mg L^−1^ CaCl_2_ (6) MS with 30 g L^−1^ sucrose, 0.5 mg L^−1^ BAP, 0.01 mg L^−1^ IBA (7) MS with 20 g L^−1^ sucrose, 0.5 mg L^−1^ BAP, 0.01 mg L^−1^ IBA (8) ½ MS with 30 g L^−1^ sucrose, 0.5 mg L^−1^ BAP, 0.01 mg L^−1^ IBA (9) ½ MS with 20 g L^−1^ sucrose, 0.5 mg L^−1^ BAP, 0.01 mg L^−1^ IBA (10) ½ MS with 30 g L^−1^ sucrose, 0.5 mg L^−1^ BAP, 0.01 mg L^−1^ IBA, 7.0 mg L^−1^ FeSO_4_·7H_2_O, 9.3 mg L^−1^ Na_2_EDTA 2H_2_O, 166 mg L^−1^ CaCl_2_ (11) ½ MS with 30 g L^−1^ sucrose, 0.25 mg L^−1^ BAP, 0.01 mg L^−1^ IBA, 7.0 mg L^−1^ FeSO_4_·7H_2_O, 9.3 mg L^−1^ Na_2_EDTA 2H_2_O, 166 mg L^−1^ CaCl_2_ (12) ½ MS with 30 g L^−1^ sucrose, 0.25 mg L^−1^ BAP, 7.0 mg L^−1^ FeSO_4_·7H_2_O, 9.3 mg L^−1^ Na_2_EDTA 2H_2_O, 166 mg L^−1^ CaCl_2_ (13) ½ MS with 30 g L^−1^ sucrose, 7.0 mg L^−1^ FeSO_4_·7H_2_O, 9.3 mg L^−1^ Na_2_EDTA 2H_2_O, 166 mg L^−1^ CaCl_2_
*Sibiraea laevigata*	(1) MS with 30 g L^−1^ sucrose, 0.5 mg L^−1^ BAP, 0.01 mg L^−1^ IBA (2) MS with 30 g L^−1^ sucrose, 0.5 mg L^−1^ BAP, 0.01 mg L^−1^ IBA, 0.1 mg L^−1^ GA_3_ (3) MS with 30 g L^−1^ sucrose, 1.0 mg L^−1^ BAP, 0.01 mg L^−1^ IBA, 0.1 mg L^−1^ GA_3_ (4) MS with 30 g L^−1^ sucrose, 1.0 mg L^−1^ BAP, 0.01 mg L^−1^ IBA, 0.2 mg L^−1^ GA_3_ (5) MS with 30 g L^−1^ sucrose, 1.0 mg L^−1^ BAP, 0.01 mg L^−1^ IBA, 0.3 mg L^−1^ GA_3_ (6) MS with 30 g L^−1^ sucrose, 0.5 mg L^−1^ BAP, 0.1 mg L^−1^ IBA, 0.1 mg L^−1^ GA_3_ (7) MS with 30 g L^−1^ sucrose, 1.0 mg L^−1^ BAP, 0.1 mg L^−1^ IBA, 0.1 mg L^−1^ GA_3_ (8) MS with 30 g L^−1^ sucrose, 0.5 mg L^−1^ BAP, 0.01 mg L^−1^ IBA, 0.2 mg L^−1^ GA_3_
*Spiraeanthus schrenkianus*	(1) MS with 30 g L^−1^ sucrose, 0.5 mg L^−1^ BAP, 0.01 mg L^−1^ IBA (2) MS with 30 g L^−1^ sucrose, 1.0 mg L^−1^ BAP, 0.01 mg L^−1^ IBA (3) MS with 30 g L^−1^ sucrose, 0.5 mg L^−1^ BAP, 0.1 mg L^−1^ IBA (4) MS with 30 g L^−1^ sucrose, 1.0 mg L^−1^ BAP, 0.1 mg L^−1^ IBA (5) MS with 30 g L^−1^ sucrose, 0.5 mg L^−1^ BAP, 0.01 mg L^−1^ IBA, 0.2 mg L^−1^ GA_3_ (6) MS with 30 g L^−1^ sucrose, 0.2 mg L^−1^ BAP, 0.02 mg L^−1^ IBA (7) MS with 30 g L^−1^ sucrose, 0.1 mg L^−1^ BAP, 0.01 mg L^−1^ IBA

* The pH of all media was 5.7. BAP—6-Benzylaminopurine; CaCl_2_—calcium chloride; Fe—iron; FeSO_4_·7H_2_O—iron sulfate heptahydrate; GA_3_—gibberellic acid; IBA—indole-3-butyric acid; MS—[64]; Na_2_EDTA 2H_2_O—ethylenediaminetetraacetic acid disodium salt.

## Data Availability

The datasets presented in the study are either included in the article or in the Supplementary Material; further inquiries can be directed to the corresponding authors.

## Supplementary Materials

The following supporting information can be downloaded at: https://www.mdpi.com/article/10.3390/plants14101526/s1, Table S1: Distribution of qualitative morphological characteristics in Rosaceae plant species.
