# Supplementary material for: Geobotanical Study and Preservation of Rare and Endangered Rosaceae Species
Source: Plants (Basel). 2025 May 19;14(10):1526. doi: 10.3390/plants14101526 (PMC12115204; doi:10.3390/plants14101526)
Supplement: Supplementary file 1 [file plants-14-01526-s001.zip › plants-3619539-supplementary.pdf]

plants

MDPI

Supplementary material

Geobotanical Study and Preservation of Rare Endangered Rosaceae Species

Table S1. Distribution of qualitative morphological characteristics in Rosaceae plant species.

Accession name/Descriptors			Quantity	Number of accession / %				
Cotoneaster karatavikus	P1	CA	9	AV (7/77.8)	YF (2/22.2)			
		SAC	9	RSC (9/100.0)				
		AGD	9	D (2/22.2)	A (6/66.7)	SD (1/11.1)		
		F	9	WF (7/77.8)	A (1/11.1)	AF (1/11.1)		
		SL	9	OLS (9/100.0)				
		SF	9	CS (4/44.4)	CCS (5/55.6)			
		CFS	9	R (2/22.2)	RG (3/33.4)	PR (2/22.2)	RB (2/22.2)	
		IS	9	A (8/88.9)	WD (1/11.1)			
	P2	PD	21	L (5/23.8)	A (16/76.2)			
		CA	21	AV (8/38.1)	YF (4/19.0)	YNF (9/42.9)		
		SAC	21	RSC (9/100.0)				
		AGD	21	SD (10/47.6)	A (11/52.4)			
		SL	21	OLS (21/100.0)				
		F	21	NF (13/61.9)	WF (2/9.5)	A (4/19.1)	AF (2/9.5)	
		SF	8	CS (3/37.5)	OLS (2/25.0)	SRC (3/37.5)		
		CFS	8	RG (3/37.5)	PG (3/37.5)	GR (1/12.5)	PR (1/12.5)	
		IS	8	A (4/50.0)	WD (4/50.0)			
	P3	PD	9	L (1/11.1)	A (8/88.9)			
		CA	9	AV (4/44.4)	YF (5/55.6)			
		SAC	9	RSC (9/100.0)				
		AGD	9	A (8/88.9)	SD (1/11.1)			
		F	9	WF (2/22.2)	A (2/22.2)	AF (5/55.6)		
		SL	9	OLS (9/100.0)				
		SF	9	CS (2/22.2)	CCS (7/77.8)			
		CFS	9	R (3/33.3)	RG (3/33.3)	PR (3/33.4)		
		IS	9	A (1/11.1)	WD (8/88.9)			
Crataegus ambigua	P1	PD	10	L (9/90.0)	A (1/10.0)			
		HA	10	L (5/50.0)	A (5/50.0)			
		SAC	10	RSC (1/10.0)	IC (9/90.0)			
		DAC	10	A (9/90.0)	SD (1/10.0)			
		F	10	WF (4/40.0)	A (6/60.0)			
		SFA	10	S (3/30.0)	A (7/70.0)			
		TQF	10	PTQ (1/10.0)	GT (9/90.0)			
	P2	CA	10	AV (4/40.0)	YF (6/60.0)			
		DAC	10	A (5/50.0)	SD (5/50.0)			
		F	10	WF (3/30.0)	A (6/60.0)	AF (1/10.0)		
	P3	CA	10	AV (3/30.0)	YF (7/70.0)			
		SAC	10	RSC (3/30.0)	IC (7/70.0)			
		DAC	10	A (9/90.0)	SD (1/10.0)			
		F	10	WF (1/10.0)	A (7/70.0)	AF (2/20.0)		

	P4	CA	10	AV (9/90.0)	YF (1/10.0)			
		DAC	10	A (6/60.0)	SD (4/40.0)			
		F	10	WF (5/50.0)	A (4/40.0)	AF (1/10.0)		
		TQF	10	A (3/30.0)	GT (7/70.0)			
Malus niedzwetzkyana	P1	SAC	2	WSC (1/50.0)	IC (1/50.0)			
		DAC	2	D (1/50.0)	A (1/50.0)			
		SL	2	ELS (1/50.0)	OLS (1/50.0)			
		RLB	2	LWP (1/50.0)	SP (1/50.0)			
		LE	2	SLE (1/50.0)	SSE (1/50.0)			
		SF	2	RSC (1/50.0)	CSF (1/50.0)			
		CFS	2	P (1/50.0)	RB (1/50.0)			
		WU	2	N (1/50.0)	A (1/50.0)			
Malus sieversii	P1	PD	35	L (14/40.0)	A (19/54.3)	T (2/5.7)		
		CA	35	OD (4/11.4)	YNF (3/8.6)	AV (20/57.1)	YF (5/14.3)	AD (3/8.6)
		HA	35	L (5/14.3)	A (16/45.7)	T (14/40.0)		
		SAC	35	PCS (3/8.6)	S (8/22.9)	RSC (18/51.4)	IC (6/17.1)	
		DAC	35	D (9/25.7)	A (19/54.3)	SD (7/20.0)		
		F	35	NF (2/5.7)	WF (24/68.6)	A (5/14.3)	AF (4/11.4)	
		SL	35	OLS (33/94.3)	ELS (2/5.7)			
		RLB	35	WPB (17/48.6)	HPB (18/51.4)			
		LE	35	SLE (1/2.9)	SSE (13/37.1)	LSE (21/60.0)		
		SF	33	RSC (27/81.9)	HSF (1/3.0)	CCS (5/15.1)		
		SFA	33	S (14/42.5)	BAS (8/24.2)	A (8/24.2)	AAS (3/9.1)	
		TQF	33	PTQ (26/78.8)	A (7/21.2)			
		CFF	33	G (30/90.9%)	Y (3/9.1)			
		FBC	33	G (21/63.6)	GY (8/24.2)	LY (2/6.1)	YR (2/6.1)	
		IS	33	A (29/87.9)	WD (4/12.1)			
		DU	33	S (12/36.4)	A (16/48.5)	DU (5/15.1)		
		WU	33	N (9/27.3)	A (23/69.7)	W (1/3.0)		
Malus sieversii	P2	PD	19	L (9/47.4)	A (6/31.6)	T (4/21.0)		
		CA	19	AV (8/42.1)	YF (2/10.5)	AD (9/47.4)		
		HA	19	L (1/5.3)	A (5/26.3)	T (13/68.4)		
		SAC	19	PCS (7/36.8)	RSC (10/52.6)	WSC (1/5.3)	IC (1/5.3)	
		DAC	19	D (10/52.6)	A (9/47.4)			
		F	19	WF (7/36.8)	A (6/31.6)	AF (6/31.6)		
		SL	19	OLS (19/100.0)				
		RLB	19	LWP (1/5.3)	WPB (10/52.6)	HPB (8/42.1)		
		LE	19	LSE (6/31.6)	SSE (7/36.8)	SE (2/10.5)	SLE (4/21.1)	
		SF	19	RSC (9/47.4)	HSF (1/5.3)	CS (1/5.3)	CSF (8/42.1)	
		SFA	19	S (1/5.3)	BAS (5/26.3)	A (10/52.6)	AAS (2/10.5)	LA (1/5.3)
		TQF	19	PTQ (10/52.6)	A (8/42.1)	GT (1/5.3)		
		FBC	19	G (10/52.6)	GY (9/47.4)			
		IS	19	A (15/78.9)	WD (4/21.1)			
		DU	19	S (5/26,3)	A (11/57,9)	DU (3/15,8)		
		WU	19	N (3/15.8)	A (13/68.4)	W (3/15.8)		
Prunus tenella	P1	CO	20	AV (7/35.0)	AFA (7/35.0)	YF (3/15.0)	YNF (3/15.0)	
		HA	20	L (6/30.0)	A (7/35.0)	T (7/35.0)		
		DAC	20	D (2/10.0)	A (10/50.0)	SD (8/40.0)		
		F	20	NF (2/10.0)	WF (18/90.0)			
		SFA	18	VS (1/5.6)	S (2/11.1)	A (15/83.3)		
		TSF	18	SWP (2/11.1)	LPS (13/72.2)	MPS (3/16.7)		
		OSS	18	ES (13/72.2)	OS (5/27.8)			
		SS	18	HO (14/77.8)	EOS (4/22.2)			
		IKC	18	LC (12/66.7)	A (6/33.3)			
Pr	P1	CA	15	AV (8/53.3)	YF (7/46.7)			

		F	15	NF (7/46.7)	WF (8/53.3)			
		HA	15	L (4/26.7)	A (11/73.3)			
		SAC	15	PCS (7/46.7)	VSP (8/53.3)			
		DAC	15	D (9/60.0)	A (6/40.0)			
		SL	15	OLS (11/73.3)	ELS (4/26.7)			
		SF	15	RSC (7/46.7)	CS (8/53.3)			
		CFS	7	BR (4/57.1)	DR (3/42.9)			
Sibiraea laevigata	P1	PD	10	L (4/40.0)	A (4/40.0)	T (2/20.0)		
		CA	10	AD (1/10.0)	AV (5/50.0)	YF (4/40.0)		
		HA	10	L (4/40.0)	T (6/60.0)			
		DAC	10	D (4/40.0)	A (5/50.0)	SD (1/10.0)		
		F	10	WF (7/70.0)	A (2/20.0)	AF (1/10.0)		
		IS	10	A (4/40.0)	WD (6/60.0)			
		CA	21	AV (19/90.5)	YF (2/9.5)			
	P2	HA	21	A (5/23.8)	T (16/76.2)			
		SAC	21	RSC (9/42.9)	OSC (9/42.9)	IC (3/14.3)		
		DAC	21	D (19/90.5)	A (2/9.5)			
		F	21	A (4/19.0)	AF (17/81.0)			
Sorbus persica	P1	PD	21	L (7/33.3)	A (1/4.8)	T (13/61.9)		
		CA	21	AV (12/57.1)	YNF (9/42.9)			
		HA	21	L (8/38.1)	A (9/42.9)	T (4/19.0)		
		SAC	21	VSP (17/81.0)	RSC (4/19.0)			
		DAC	21	D (3/14.3)	A (15/71.4%)	SD (3/14.3)		
		SL	21	OLS (17/81.0)	ELS (4/19.0)			
		RLB	21	WPB (8/38.1)	HPB (13/61.9)			
		LE	21	LLE (3/14.3)	SLE (1/4.8)	DLE (17/81.0)		
	P2	CA	5	AV (1/20.0)	YNF (4/80.0)			
		HA	5	L (3/60.0)	T (2/40.0)			
		SAC	5	VSP (4/80.0)	IC (1/20.0)			
		DAC	5	A (3/60.0)	SD (2/40.0)			
		SL	5	OLS (4/80.0)	ELS (1/20.0)			
Spiraeanthus schrenckianus	P1	PD	12	L (9/75.0)	A (3/25.0)			
		CA	12	AV (11/91.7)	YF (1/8.3)			
		HA	12	L (9/75.0)	A (2/16.7)	T (1/8.3)		
		DAC	12	D (2/16.7)	A (7/58.3)	SD (3/25.0)		
		F	12	WF (3/25.0)	A (5/41.7)	AF (4/33.3)		
		SFA	12	A (8/66.7)	AAS (4/33.3)			
	P2	PD	8	T (7/87.5)	A (1/12.5)			
		HA	8	L (7/87.5)	A (1/12.5)			
		SAC	8	RSC (6/75.0)	SCS (2/25.0)			
		DAC	8	D (7/87.5)	A (1/12.5)			
		SFA	8	S (1/12.5)	A (4/50.0)	AAS (2/25.0)	BAS (1/12.5)	

Descriptors: Accession growth density (**AGD**); Condition of the accession (**CA**); Color of the fruit flesh (**CFF**); Color of the fruit skin (**CFS**); Density of the accession crown (**DAC**); Depth of the umbilicus (**DU**); Fruiting (**F**); Fruits Basic color (**FBC**); Height of the accession (**HA**); Intensity of the kernel color (**IKC**); Integrity of the skin (**IS**); Leaf edge (**LE**); Opening of the shell along the seam (**OSS**); Population density (**PD**); Reverse side of the leaf blade (**RLB**); Shape of the accession crown (**SAC**); Shape of the fruit (**SF**); Size of the accession fruit (**SFA**); Shape of the leaf (**SL**); Strength of the shell (**SS**); Taste quality of fruits (**TQF**); Texture of the outer shell of the fruit (**TSF**); Width of the umbilicus (**WU**).

Morphological features: Average (**A**); Above average size (**AAS**); Adult diseased (**AD**); Abundant fruiting (**AF**); Adult fruitful accession (**AFA**); Adult viable (**AV**); Below average size (**BAS**); Bright red (**BR**); Conical crown shape (**CCS**); Cylindrical shape (**CS**); Conical fruits shape (**CSF**); Dense (**D**); Dentate leaf edge (**DLE**); Dark red (**DR**); Deep umbilicus (**DU**); Elliptic leaf shape (**ELS**); Easy to open shell (**EOS**); Excellent sealing (**ES**); Green (**G**); Greenish-red (**GR**); Good taste (**GT**); Greenish-yellow (**GY**); Hard to open (**HO**); Lamina Heavily pubescent leaf blade (**HPB**); Heart-shape of the fruits (**HSF**); Irregular crown (**IC**); Large (**L**); Low accession (**LA**); Light color (**LC**); Lobed leaf

edge (**LLE**); Low-porous shell (**LPS**); Largely serrated leaf edge (**LSE**); Lamina without pubescence (**LWP**); Light yellow (**LY**); Medium-porous shell (**MPS**); Narrow (**N**); No fruiting (**NF**); Old decrepit (**OD**); Ovate leaf shape (**OLS**); Open shell (**OS**); Oval shape crown (**OSC**); Pink (**P**); Pyramidal crown shape (**PCS**); Pinkish-green (**PG**); Pinkish-red (**PR**); Poor taste qualities (**PTQ**); Red (**R**); Red-brown (**RB**); Red-green (**RG**); Round shape crown (**RSC**); Short round conical (**SRC**); Small (**S**); Spreading crown shape (**SCS**); Sparse density (**SD**); Serrated leaf edge (**SE**); Small-lobed leaf edge (**SLE**); Slightly pubescent (**SP**); Small-serrated leaf edge (**SSE**); Shell without pores (**SWP**); Tall (**T**); Very small (**VS**); Vase shape crown (**VSC**); Wide (**W**); Without damage (**WD**); Weak fruiting (**WF**); Weakly pubescent leaf blade (**WPB**); Weeping shape crown (**WSC**); Yellow (**Y**); Young fruitful (**YF**); Young non-fruitful (**YNF**); Yellow-red (**YR**).
P1-P4 – Number of populations.
